# A novel combination of multiple primary carcinomas: Urinary bladder transitional cell carcinoma, prostate adenocarcinoma and small cell lung carcinoma- report of a case and review of the literature

**DOI:** 10.1186/1477-7819-3-51

**Published:** 2005-07-26

**Authors:** Anastassios V Koutsopoulos, Konstantina I Dambaki, George Datseris, Elpida Giannikaki, Marios Froudarakis, Efstathios Stathopoulos

**Affiliations:** 1 Department of Pathology, Heraklion University Hospital, Greece; 2 Department of Pneumonology, Heraklion University Hospital, Greece

## Abstract

**Background:**

The incidence of multiple primary malignant neoplasms increases with age and they are encountered more frequently nowadays than before, the phenomenon is still considered to be rare.

**Case presentation:**

We report a case of a man in whom urinary bladder transitional cell carcinoma, metachronous prostate adenocarcinoma and small cell lung carcinoma were diagnosed within an eighteen-month period. The only known predisposing factor was that he was heavy smoker (90–100 packets per year). The literature on the phenomenon of multiple primary malignancies in a single patient is reviewed and the data is summarized.

**Conclusion:**

It is important for the clinicians to keep in mind the possibility of a metachronous (successive) or a synchronous (simultaneous) malignancy in a cancer patient. It is worthy mentioning this case because clustering of three primary malignancies (synchronous and metachronous) is of rare occurrence in a single patient, and, to our knowledge, this is the first report this combination of three carcinomas appearing in the same patient.

## Background

The phenomenon of multiple primary malignant neoplasms in the same individual was described firstly by Billroth at the end of the 19^th ^century [1]. Since then, several cases of double or even triple primary malignant neoplasms have been reported. It is believed that multiple primary malignant neoplasms now occur more frequently than before. Although, not uncommon, they occur more often in elderly patients, as the incidence of malignancies increases with age. The diagnosis of second primary neoplasms is rising as a result of prolonged survival of patients treated for previous malignancy with alkylating agents, topoisomerase II inhibitors, and/or radiotherapy[2]. A review of the recent literature indicates clearly that they appear more frequently in the upper digestive tract, respiratory system, head and neck region, or urogenital system; the reported incidence ranges from 2% to 10% [3].

In this report we present a patient who developed primary bladder carcinoma and metachronous prostate and small cell lung carcinoma (SCLC) within an eighteen-month period. This combination of multiple primary carcinomas, to our knowledge, has never been reported in the literature.

## Case presentation

**Figure 1:**
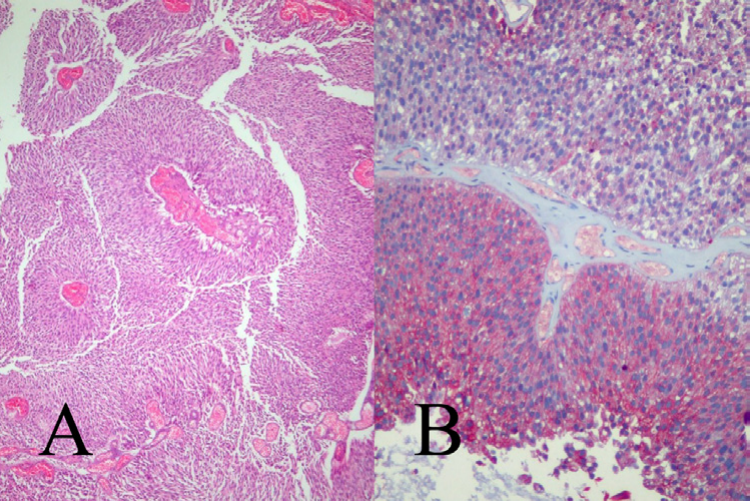
Microscopically, the extracted urinary bladder tissue particles proved to be pieces of papillary transitional cell carcinoma grade II [**A) **hematoxylin and eosin × 40] and immunohistochemically they expressed cytokeratin 7 [**B) **cytokeratin 7 × 100].

**Figure 2:**
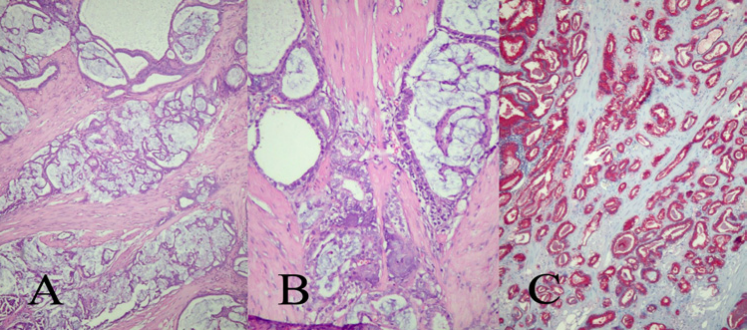
Histologically, in most of the prostate tissue fragments were recognized areas of, partially mucinous, adenocarcinoma of the prostate, grade II-III (**A. **hematoxylin and eosin × 40, **B**: hematoxylin and eosin × 100). The tumor cells were strongly positive for PSA (**C**: PSA × 40).

**Figure 3:**
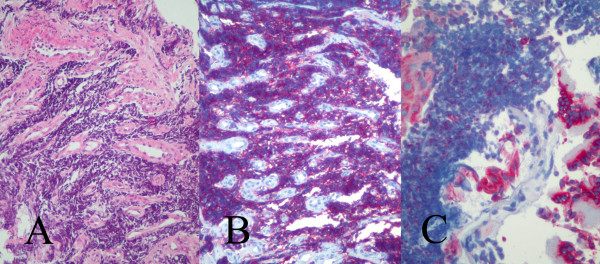
The bronchial mucosa showed extensive invasion from small blue round cells (**A**: hematoxylin and eosin × 100) that were positive for the neuroendocrine marker CD56 (**B**: × 200) and pan-cytokeratin (**C**: × 200).

A 75-year old ex-smoker (90–100 packet per year) underwent a transurethral resection of urinary bladder papilloma in February 2002. The histology of resected specimen was papillary transitional cell carcinoma grade II (Figure 1A). The tumor cells were positive for cytokeratin 7 (Figure 1B) and negative for cytokeratin 20. There were no muscle fibers in the examined tissue. The ultrasound examination of the urogenital system revealed nodular hyperplasia of the prostate. The tumor clinical stage according to the American Cancer Committee U.I.C.C. (1992) was Ta. Patient's cancer relapsed at the end of the same year and he underwent a programmed transurethral resection of the tumor, which proved to be papillary transitional cell carcinoma grade I-II. No lamina propria or muscle invasion was detected. The patient was also treated with intracystic infusion of bacille Calmette-Guerin (BCG). Ten days later, because of urine retention, he underwent transurethral resection of the prostate. Multiple tissue fragments of total dimensions 4.5 × 3.5 × 2.2 cm were examined histologically. Seven out of the 10 examined slides revealed foci of partially mucinous (Figure 2A) adenocarcinoma of the prostate (the greatest measured focus was 8.5 mm in maximum diameter), Gleason grade II-III and Gleason score 5 (Figure 2A, B). Immunohistochemical study was performed and showed strong positivity for Prostate Specific antigen (PSA) (Figure 2C) whereas; no expression of carcinoembryonic antigen (CEA) was detected in tumor cells. These findings confirmed the diagnosis of primary prostate adenocarcinoma. The tumor's stage according to the 1997 TNM staging system of prostatic adenocarcinoma was T1b. Serum prostate specific antigen (PSA) levels were elevated (9 ng/mL) before surgery. No additional surgical treatment was given and at follow-up visits prostate specific antigen (PSA) levels measurement and intracystic injection of BCG was performed. In September of the same year, due to progressively worsening dyspnea a computed tomography was performed that revealed a mediastinal mass in conjunction to the right lung hilum and to the right main bronchus with maximum diameter of 9 cm. Bronchoscopy showed a large mass which invaded the right main bronchus mucosa and extended to the carina. Histology of the bronchial mucosal sample showed infiltration of lamina propria by malignant cells (Figure 3A). Their immunophenotype was: CD56 (+) (Figure 3B), Pan-Cytokeratin (paranuclear dot stain positivity) (Figure 3C) and Leukocyte Common Antigen negative. Combining the morphological and the immunohistochemical results, we concluded that the patient was suffering from small cell lung carcinoma (SCLC). The patient's stage was IIIB. Ten days after the diagnosis was confirmed, the patient underwent the first cycle of chemotherapy (Cisplatin and Vepesid), during which he died from cardiac arrest due to chemotherapy toxicity.

## Discussion

We report a patient who developed three histologically distinct malignancies, i.e. primary bladder carcinoma and metachronous prostate and SCLC within an eighteen-month period. There are several predisposing or causal factors for each malignancy. For our patient there was only one common causal factor, the fact that he was a heavy smoker (90–100 packets per year). No other predisposing factor or a family history was found that might have contributed to the development of these three malignancies. The presence of bladder and prostate carcinomas in the same patient is not a rare event. Chun [3] reported that the rate of bladder carcinoma in patients with prostate carcinoma is eighteen times higher (p < 0,01) and the rate of prostate carcinoma in those with bladder carcinoma is nineteen times higher (p < 0,01) than expected. Although bladder and prostate carcinoma can coexist in the same individual frequently enough, the rare event is the appearance of a third malignancy. There is a case report by Rovinescu *et al *[4] referring to a patient with three primary malignancies. The first tumor was a clear cell carcinoma of the kidney, which was followed by a transitional cell carcinoma of the bladder and then by a distinct adenocarcinoma of the prostate. More recently, in 2003, Satoh *et al *[5] also reported the same combination of multiple primary malignancies in a patient. Our case is the first one of an individual having these two primary malignancies of the urogenital system and another tumor of the lower respiratory tract.

Table 1 summarizes the cases with three or more primary malignancies. As can be easily seen, although the appearance of three primary malignancies in one patient is not very common, should not be considered such a rare event.

**Table 1:** There are summarized the cases of triple or more malignancies, the first author, journal, year of publication and combination of neoplasms.

	**Year**	**Author**	**1st Malignancy**	**2nd Malignancy**	**3rd Malignancy**	**4th Malignancy**	**5th Malignancy**
1	1949	Crail H.W [6]	Thyroid Carcinoma	Rectal Carcinoma	Medulloblastoma		
2	1974	Hamoudi A.B.[7]	Colon Carcinoma	Thymus Carcinoma	Skin Carcinoma	Astrocytoma G3	
3	1975	Ohsato K. [8]	Colon Carcinoma	Astrocytoma G3	Duodenal Carcinoma		
4	1976	Kawanami K. [9]	Ileum Carcinoma	Glioblastoma	Colon Carcinoma		
5	1976	Rovinescu I.[4]	Clear Cell Carcinoma Of Kidney	Transitional Cell Carcinoma Of Bladder	Prostate Carcinoma		
6	1979	Itoh H.[10]	Colon Carcinoma	Stomach Carcinoma	Astrocytoma G3		
7	1979	Mullen J.L.[11]	Hodgkin' Disease	Squamous Cell Carcinoma Of Larynx	Squamous Cell Carcinoma In Esophagus		
8	1979	Pinel J.[12]	7 Squamous Cell Carcinomas In 9 Years				
9	1980	Cohen C.[13]	Multiple Cutaneous Squamous Cell Carcinomas	Multiple Cutaneous Basal Cell Carcinomas	Diffuse Poorly Differentiated Lymphocytic Lymphoma		
10	1982	Friedman C.D. [14].	Breast Carcinoma	Colon Carcinoma	Glioblastoma In Brain		
11	1983	Li F.P.[15]	Colon Carcinoma	Astrocytoma G3	Leukemia		
12	1984	Haibach H.[16]	Thyroid Carcinoma	Renal Carcinoma	Duodenal Carcinoma		
13	1985	Alessi E.[17]	Multiple Sebaceous Tumors	Keratoacanthoma	3 Primary Adenocarcinomas Of Colon		
14	1985	Kobayashi T. [18]	Uterus Carcinoma	Stomach Carcinoma	Breast Carcinoma	Glioblastoma In Brain	
15	1985	Megighian D.[19]	Squamous Cell Carcinoma Of Parotid	Squamous Cell Carcinoma Of Tongue	Squamous Cell Carcinoma Of Soft Palate	Squamous Cell Carcinoma Of Larynx	Squamous Cell Carcinoma Of Hypopharynx
16	1985	Staren E.D.[20]	Carcinoma Of Larynx	Carcinoma Of Floor Of Mouth	Dual Primary Bronchogenic Carcinomas		
17	1986	Craig D.M.[21]	Squamous Cell Carcinoma Of The Floor Of The Mouth	Adenocarcinoma Of Lung	Squamous Cell Carcinoma Of Larynx	Squamous Cell Carcinoma Of The Tongue	
18	1986	Ogasawara K.[22]	Breast Carcinoma	Breast Carcinoma	Lung Carcinoma	Glioblastoma In Brain	Thyroid Carcinoma
19	1987	Hayashi K.[23]	Colon Carcinoma	Rectal Carcinoma	Glioblastoma In Brain		
20	1987	Kobayashi T.[24]	Uterus Carcinoma	Stomach Carcinoma	Glioblastoma In Brain		
21	1988	Ohi H. [25]	Skin Carcinoma	Medulloblastoma	Thyroid Carcinoma		
22	1991	Baigrie R.J.[26]	7 Primary Carcinomas				
23	1991	Solan M.J.[27]	Two Breast Carcinomas	Thyroid Carcinoma	Multiple Skin Carcinomas		
24	1992	Melkert P.W. [28]	Squamous Cell Carcinoma Of Skin	Squamous Cell Carcinoma Of Vulva	Squamous Cell Carcinoma Of Vagina	Squamous Cell Carcinoma Of Anus	Squamous Cell Carcinoma Of Cervix Uteri
25	1992	Marcos Sanchez F. [29]	Colon Carcinoma	Renal Carcinoma	Breast Carcinoma		
26	1993	Brugieres L. [30]	Soft Tissue Tumor	Brain Tumor	Thyroid Carcinoma	Breast Carcinoma	
27	1993	Kikuchi T. [31]	Glioblastoma	Colon Carcinoma	Colon Carcinoma		
28	1993	Shiseki M.[32]	Skin Carcinoma	Colon Carcinoma	Glioblastoma In Brain		
29	1994	Bumpers H.L.[33]	Squamous Cell Carcinoma Of Larynx	Squamous Carcinoma Of Lung	Adenocarcinoma Of Breast	Adenocarcinoma Of Colon	
30	1994	Nishihara K. [34]	Papillary Adenocarcinoma Of Papilla Of Vater	Papillary Adenocarcinoma Of Common Bile Duct	Papillary Adenocarcinoma Of Pancreas		
31	1995	Angeli-Besson C. [35]	Chronic Myeloid Leukemia, Multiple Squamous Cell Carcinomas				
32	1996	Hayashi T.[36]	Squamous Cell Carcinoma In Soft Palate	Squamous Cell Carcinoma In Larynx	Squamous Cell Carcinoma In Esophagus		
33	1996	Nagane M.[37]	Tubular Adenocarcinoma Of Stomach	Transitional Cell Carcinoma Of Bladder	Glioblastoma In Brain		
34	1996	Nagane M. [37]	Papillary Adenocarcinoma Of Lung	Adenocarcinoma Of Rectum	Glioblastoma In Brain		
35	1997	Potzsch C.[38]	Breast Carcinoma	Small Cell Lung Carcinoma	Renal Cell Carcinoma	Acute Myelomonocytic Leukemia	
36	1997	Shan L.[39]	14 Foci Of Primary SCC, Esophagus, Oral Floor, Soft Palate, Uvula, Lingual Radix, Piriform Recess, Hypopharynx, Trachea, Lingual Body				
37	1999	Cribier B. [40]	Eccrine Porocarcinoma	Tricholemmal Carcinoma	Multiple Squamous Cell Carcinomas		
38	1999	Ramsay H.M.[41]	Acute Myeloid Leukemia	Chronic Lymphocytic Leukemia	Basal Cell Carcinomas		
39	1999	Schon M.P.[42]	Basal Cell Carcinomas	Hairy Cell Leukemia	Basal Cell Carcinomas		
40	2000	Beswick S.J.[43]	Basal Cell Carcinomas	Malignant Melanoma In Situ	Basal Cell Carcinomas		
41	2001	Mukai [44]	Stomach Carcinoma	Duodenal Carcinoma	Esophageal Cancer	Renal Cancer	Colon Carcinoma In Situ
42	2003	Satoh H.[5]	Carcinoma Of Kidney	Transitional Cell Carcinoma Of Bladder	Prostate Carcinoma		

Additionally, studying the existing bibliography, we noticed that there is a little confusion regarding the terms used, such as synchronous, simultaneous and metachronous or successive neoplasms. All of these words have to do with the time that the neoplasms are discovered and have nothing to do with the time of their genesis. The word synchronous is a Greek one that should refer to neoplasms appearing in the same time. It is synonymous to the word simultaneous and they are interchangeable. Metachronous (meta- means after and -chronous is the time) is also a Greek word referring to a neoplasm that is discovered while there is already a known neoplasm in the same patient. The word successive could be used equally to metachronous.

## Conclusion

Summarizing, it is important for the clinicians to keep in mind that the appearance of another tumor in a patient suffering from cancer could be either a metastasis or another malignancy and should always investigate the possibility of a metachronous (successive) or a synchronous (simultaneous) malignancy. Moreover, the combination of the three different neoplasms (bladder, prostate and SCLC) in one patient, to the best of our knowledge, has never been reported before.

## Competing interests

The author(s) declare that they have no competing interests.

## Authors' contributions

**KAV **wrote the original manuscript and performed histopathological evaluation of the lung lesion.

**DKI **participated in the writing of the original manuscript and prepared photomicrographs.

**DG **performed histopathological evaluation of the urinary bladder lesion.

**GE **performed histopathological evaluation of the prostate lesion.

**FM **performed bronchoscopy and patient's management.

**SE **prepared requested revisions of the manuscript.
